# Tissue-specific diffusion abnormalities and localized free-water reductions in hypertension despite no detectable group difference in the DTI-ALPS index

**DOI:** 10.3389/fmed.2026.1867342

**Published:** 2026-08-12

**Authors:** Taipeng Zeng, Xiaoyang Wang, Wenhui Xu, Zhifeng Huang, Hui Xiong, Kanlin Lin, Hao Huang, Jianfeng Chu, Shangwen Xu, Liyuan Fu

**Affiliations:** 1 Fuzong Teaching Hospital, Fujian University of Traditional Chinese Medicine (900th Hospital), Fuzhou, China; 2 College of Integrative Medicine, Fujian University of Traditional Chinese Medicine, Fuzhou, China; 3 The 910th Hospital of the People's Liberation Army Joint Logistics Support Force, Quanzhou, China; 4 Fujian Academy of Chinese Medical Sciences, Fuzhou, China

**Keywords:** cortical gray matter, diffusion tensor imaging, essential hypertension, free-water elimination, microstructural abnormalities, white matter

## Abstract

**Background:**

Essential hypertension (HTN) is an established risk factor for cerebral small vessel disease and cognitive decline. Although conventional diffusion tensor imaging (DTI) has identified white matter (WM) abnormalities in HTN, these metrics are often confounded by extracellular free-water (FW) contamination and cannot distinguish tissue microstructural abnormalities from extracellular fluid shifts. This study applied a free-water elimination (FWE) model in combination with the diffusion tensor imaging analysis along the perivascular space (DTI-ALPS) index to investigate tissue-specific microstructural alterations, extracellular fluid shifts, and glymphatic function in both WM and cortical gray matter (GM).

**Methods:**

Single-shell diffusion magnetic resonance imaging (MRI) data were acquired from 30 patients with HTN and 30 age- and sex-matched healthy controls (HCs). Tissue-specific DTI metrics, including free-water-corrected fractional anisotropy (FAt) and free-water-corrected mean diffusivity (MDt), as well as FW fractional volume maps, were derived. The DTI-ALPS index was calculated to assess glymphatic clearance. Tract-based spatial statistics (TBSS) and gray matter-based spatial statistics (GBSS) were used to evaluate between-group differences.

**Results:**

The HTN group showed no statistically significant difference in the DTI-ALPS index compared with the HC group (*p* > 0.05). However, alongside conventional diffusion abnormalities, patients with HTN exhibited widespread reductions in FAt and increases in MDt across both WM and GM skeletons. In addition, HTN patients showed a localized reduction in FW fractional volume, predominantly in the corpus callosum. Systolic blood pressure (SBP) was negatively correlated with conventional FA in white matter, and with both FA and FAt in cortical GM across extensive regions. No significant correlations were found with Mini-Mental State Examination (MMSE) scores.

**Conclusions:**

These cross-sectional findings suggest that, in HTN, tissue-specific diffusion abnormalities and localized fluid shifts are detectable even in the absence of statistically significant group differences in the DTI-ALPS index. This divergence indicates that FWE metrics may serve as sensitive neuroimaging indicators for evaluating hypertensive brain involvement.

## 1 Introduction

Essential hypertension (HTN) is a major global risk factor for cardiovascular disease, stroke, and cognitive impairment (1–4). The brain is a primary target organ of hypertensive injury, as chronic hemodynamic stress may induce microvascular remodeling, endothelial dysfunction, and blood-brain barrier (BBB) disruption (5–7). Instead of a vague cascade, these vascular insults directly compromise the brain parenchyma, leading to subtle neuroinflammation, myelin alterations, and microstructural abnormalities (8, 9). To capture these insidious structural damages *in vivo*, advanced neuroimaging techniques have become indispensable. While conventional diffusion tensor imaging (DTI) has been widely used to demonstrate *in vivo* microstructural compromises in HTN, typically presenting as reduced fractional anisotropy (FA) and increased mean diffusivity (MD) (10–12), a critical methodological limitation persists. Conventional DTI metrics are inherently confounded by partial volume effects from the extracellular space (13, 14), making it challenging to decipher whether the observed FA reductions stem from true neural tissue injury or merely from fluid shifts in the extracellular environment (15–18).

To address this ambiguity, the free-water elimination (FWE) model (19) was developed to distinctively separate the diffusion signal into tissue-specific and extracellular fluid (FW) compartments. Although FWE has been predominantly applied in advanced neurodegenerative conditions, where elevated FW typically reflects atrophy-related space expansion or vasogenic edema (20–22), its application in hypertensive brain involvement may reveal a fundamentally different trajectory (23, 24). Under chronic, mild microvascular hypoperfusion, cellular stress might trigger reactive astrogliosis or subtle fluid redistribution, potentially restricting the extracellular space and paradoxically reducing FW (25, 26). Furthermore, while the majority of DTI studies in HTN have focused exclusively on major white matter (WM) tracts (27, 28), the cortical microenvironment is also highly susceptible to vascular injury, necessitating a comprehensive evaluation of both WM and gray matter (GM) microstructural reorganization.

Concurrently with these parenchymal changes, the glymphatic system, a brain-wide network mediating perivascular fluid and metabolic waste clearance, has attracted increasing attention in hypertensive research (29, 30). The DTI analysis along the perivascular space (DTI-ALPS) index has emerged as a robust, noninvasive proxy for macroscale glymphatic function (31–33). While a reduced ALPS index is consistently associated with advanced hypertensive cerebral small vessel disease (34), and is increasingly investigated as a potential early biomarker for preclinical cognitive vulnerability (35–37), the precise temporal dynamics essential HTN remain largely elusive. It remains to be clarified whether macroscale glymphatic dysfunction acts as a primary driver of brain injury, or whether localized parenchymal fluid shifts and tissue-specific damage might be detectable even when perivascular clearance remains functionally compensated.

Therefore, in this observational study, we utilized single-shell diffusion MRI, the FWE model, and ALPS analysis to jointly investigate microstructural alterations, extracellular fluid shifts, and glymphatic clearance across both WM and GM in patients with essential HTN. Specifically, we aimed to: (1) determine whether tissue-specific microstructural abnormalities remain detectable after accounting for FW contamination; (2) characterize the spatial distribution of FW changes; (3) evaluate the integrity of macroscale glymphatic function using the ALPS index and explore its relationship with parenchymal fluid alterations; and (4) assess the associations of these neuroimaging metrics with systolic blood pressure (SBP) and cognitive performance. We postulated that FWE-derived metrics would reveal significant tissue-specific diffusion abnormalities and localized fluid shifts. We further hypothesized that these microstructural vulnerabilities, alongside potential alterations in glymphatic clearance, would exhibit strong associations with elevated SBP, thereby providing novel, noninvasive insights into the pathophysiological events in the hypertensive brain.

## 2 Methods and materials

### 2.1 Subjects

The study was approved by the Biomedical Ethics Committee of the 900th Hospital of the Joint Logistics Support Force (Approval Number: 2016-009), and all participants provided written informed consent. Participants were prospective recruited between July 2016 and December 2025. A total of 30 patients with HTN and 30 healthy controls (HCs) were recruited using frequency matching for age and sex. The inclusion criteria for HTN patients were based on the 2018 ESC/ESH Guidelines for the Management of Arterial Hypertension (38), defined as a clinical SBP ≥ 140 mmHg and/or diastolic blood pressure (DBP) ≥ 90 mmHg, or the current use of antihypertensive medications. All participants underwent structural and diffusion MRI. Exclusion criteria for all participants included: (1) a history of stroke, dementia, schizophrenia, epilepsy, Parkinson's disease, bipolar disorder, or any other major neurological or psychiatric disorder; (2) use of medications known to affect cognition; (3) claustrophobia; (4) secondary HTN; (5) end-stage cardiac disease; (6) renal failure (including dialysis); (7) diabetes mellitus; and (8) atrial fibrillation. Routine clinical MRI sequences were performed to exclude macroscopic intracranial pathological findings, such as brain tumors, previous cerebral hemorrhage, and congenital anomalies. All routine clinical scans were independently evaluated by two experienced radiologists who were blinded to group allocation. Participants with visually severe, confluent white matter lesions were excluded to minimize confounding from advanced cerebral small vessel disease. A detailed participant selection and exclusion flow is provided in Supplementary Figure S1.

### 2.2 Clinical, cognitive, and laboratory assessments

All participants underwent a comprehensive clinical evaluation. Blood pressure (SBP and DBP) measurements strictly adhered to standard protocols and were recorded as the average of three separate measurements. For laboratory analysis, fasting venous blood samples were collected from all subjects following an overnight fast of at least 8 h to measure relevant serum biochemical and endocrine biomarkers (e.g., sex hormones and standard blood panels). Additionally, cognitive function was assessed by a trained neuropsychologist using the mini-mental state examination (MMSE) to evaluate global cognitive status across domains such as orientation, attention, recall, and language.

### 2.3 Image acquisition and processing

MRI data were acquired using a 3.0 T Trio Tim superconducting MRI scanner (Siemens Healthcare, Erlangen, Germany) equipped with a 12-channel phased-array head coil. To minimize head motion artifacts, foam pads were placed within the head coil, and earplugs were provided for hearing protection. Three-dimensional T1-weighted images (T1WI) were obtained using a fast gradient-echo sequence with the following parameters: repetition time (TR) = 1,900 ms, echo time (TE) = 2.52 ms, slice thickness = 1 mm, field of view (FOV) = 256 mm × 256 mm, and matrix size = 256 × 256. Diffusion-weighted images (DWI) were acquired using a spin-echo echo-planar imaging (SE-EPI) sequence. The diffusion-sensitizing gradients were applied along 64 non-collinear directions with a b-value of 1,000 s/mm^2^, along with one non-diffusion-weighted image (*b* = 0 s/mm^2^). Other parameters included: TR = 7,200 ms, TE = 104 ms, slice thickness = 2.5 mm, 49 axial slices, FOV = 256 mm × 256 mm, matrix size = 128 × 128, flip angle = 90°, parallel imaging acceleration factor (iPAT) = 2, and acquisition bandwidth = 1,396 Hz/Px. The phase-encoding direction was anterior-to-posterior (AP). The acquisition coverage extended from the skull base to the vertex. Total acquisition time: 8 min 11 s.

All DWI data were preprocessed using MRtrix3 (https://www.mrtrix.org) (39) and FMRIB Software Library (FSL, https://fsl.fmrib.ox.ac.uk/fsl/fslwiki/) (40). The preprocessing steps included denoising, removal of Gibbs ringing artifacts, eddy-current and subject movement correction using FSL's eddy tool, and B1 field inhomogeneity correction. Since reverse phase-encoded images were not acquired, susceptibility-induced geometric distortion correction was performed using a non-linear registration-based approach by aligning the *b* = 0 image to the subject's structural T1WI (using FSL's epi_reg command to estimate the boundary-based registration, followed by applywarp to resample the diffusion data). Quantitative motion metrics were extracted to ensure no participant exceeded the predefined motion threshold of 2.5 mm translation or 2.5 degrees rotation. Estimated total intracranial volume (TIV) was calculated from the 3D T1WI using FreeSurfer (v8.1.0, https://surfer.nmr.mgh.harvard.edu/) (41). For structural image processing, bias field correction was performed using N4BiasFieldCorrection, followed by robust brain extraction using SynthStrip. Tissue segmentation was conducted using the Atropos algorithm within Advanced Normalization Tools (ANTs, http://stnava.github.io/ANTs/) (42) with MNI152 FAST priors to accurately delineate GM, WM, and cerebrospinal fluid (CSF) probability maps.

Following preprocessing, conventional DTI maps were generated to derive FA, MD, axial diffusivity (AD), and radial diffusivity (RD). To assess glymphatic system function, the DTI-ALPS index was calculated. Following established protocols, diffusivity values were extracted from the projection fibers (corona radiata) and association fibers (superior longitudinal fasciculus) at the level of the lateral ventricle body in the native space. Spherical regions of interest (ROIs) measuring 5 mm in diameter were manually placed. To ensure measurement reliability and minimize subjective bias, ROI placement was performed independently by two trained researchers who were strictly blinded to the participants' clinical and group assignments. The final ALPS index for each subject was determined by averaging the measurements obtained from both independent raters. Additionally, indices from the left and right hemispheres were calculated separately and averaged. The ALPS index was defined mathematically as:


$$\begin{array}{l} ALPS=\frac{D_{xxproj}+D_{xxassoc}}{D_{yyproj}+D_{zzassoc}} \end{array}$$

where *D*_xx_, *D*_yy_, and *D*_zz_ denote the principal diffusivities along the *x, y*, and *z* axes, respectively.

In parallel, a bi-tensor model was fitted to the DWI data using the FreeWaterTensorModel in the open-source diffusion imaging in Python (DIPY, v1.7.0; Python Software Foundation (PSF), Beaverton, OR, USA) software package (43). Because single-shell free-water fitting is intrinsically underdetermined, the DIPY implementation employs spatial regularization to stabilize parameter estimation and constrain the FW fraction. The fitting algorithm was initialized using a standard non-linear least squares approach to provide baseline estimates. To prevent biologically implausible results, parameter constraints were applied within the algorithm, bounding the FW fractional volume between 0 and 1, and restricting tissue diffusivities to strictly positive values. The algorithm iterated until standard convergence criteria (change in parameters < 0.0001) were met. The diffusivity of the FW compartment was fixed at the physical constant of water at body temperature (3.0 × 10^−3^ mm^2^/s). Model fits were visually inspected, and goodness-of-fit maps were evaluated to monitor the frequency of failed or boundary-valued estimates (which were < 1%). This procedure generated maps of FW fractional volume and FW-corrected tissue metrics, including FW-corrected FA (FAt), FW-corrected MD (MDt), FW-corrected AD (ADt), and FW-corrected RD (RDt). For qualitative quality-control visualization, a representative native-space *b* = 0 image and the corresponding free-water fraction map are provided in Supplementary Figure S2.

### 2.4 Voxel-wise analysis

Voxel-wise statistical analyses were performed to regionally map significant between-group differences across all nine diffusion parameters (FA, MD, AD, RD, FAt, MDt, ADt, RDt, and FW). This was achieved using tract-based spatial statistics (TBSS) for white matter (WM) and gray matter-based spatial statistics (GBSS) for cortical GM.

TBSS was carried out using standard procedures in FSL (44). Individual FA maps were non-linearly registered to the standard Montreal Neurological Institute (MNI152) space using FSL's FNIRT tool. A study-specific mean FA image was then generated and thinned to create a mean FA skeleton representing the centers of major WM tracts. This skeleton was thresholded at FA > 0.20 to exclude peripheral tracts and GM regions. The aligned diffusion maps (both conventional and FW-corrected metrics) of each subject were subsequently projected onto this mean FA skeleton for voxel-wise statistics.

For GM analysis, a customized GBSS pipeline was implemented. As described in recent methodological frameworks (45–47), GBSS adapts the TBSS approach to overcome partial volume contamination within cortical microstructures by skeletonizing the GM and projecting diffusion metrics from the most probable local GM voxel onto the skeleton. Each subject's T1WI was non-linearly registered to the MNI152 template using ANTs (antsRegistrationSyN), and the resulting transformations were applied to warp the GM probability maps into MNI space. A group-level GM skeleton was constructed based on the voxel-wise median of the GM probability maps across all subjects. Specifically, individual warped GM probability maps were merged into a 4D image to compute the median map. This median map was thresholded at a strict GM probability of 0.2 to generate a continuous GM skeleton that captures the structural core of the cortical GM.

For multimodal integration, the non-diffusion-weighted (*b* = 0) image was extracted from each subject's diffusion dataset and affine-aligned to their corresponding structural T1WI using FSL's epi_reg, which employs boundary-based registration (BBR). Diffusion-derived scalar maps were subsequently aligned to the T1 space using these estimated transformations, and then warped to MNI space utilizing the previously computed T1-to-MNI non-linear warp fields.

To accurately project the diffusion metrics onto the GM skeleton, a localized maximum-probability search algorithm was applied. For each voxel on the GM skeleton, the algorithm searched within a restricted spatial neighborhood (maximum search radius of 1 voxel) perpendicular to the local surface and identified the voxel with the highest GM probability. The diffusion metric value at this exact location, mapped using nearest-neighbor interpolation to preserve the raw intensity values, was then assigned to the skeleton voxel. As part of quality control, individual FA maps projected onto the TBSS and GBSS skeletons were visually inspected for gross misregistration or projection errors. Complete subject-level displays of the skeleton-projected FA maps for all 60 analyzed participants are provided in Supplementary Figure S3. This crucial step restricts the projection to voxels most likely belonging to gray matter, effectively mitigating partial volume contamination from adjacent WM or CSF. Finally, the skeletonized diffusion metric maps from all subjects were merged into 4D datasets for group-level statistical comparisons.

### 2.5 Statistical analysis

Demographic, clinical, cognitive, and laboratory characteristics were compared between groups using independent-samples *t* tests for normally distributed continuous variables and Mann–Whitney *U* tests for non-normally distributed variables. Categorical variables were compared using chi-square tests. In addition, group differences in the DTI-ALPS index were assessed using analysis of covariance (ANCOVA), with age, sex, and TIV included as covariates. A two-tailed *p* value < 0.05 was considered statistically significant.

Voxel-wise statistical analysis across the WM and GM skeletons was performed using the randomize tool in FSL. A general linear model framework was applied to evaluate group differences in diffusion metrics between HTN patients and HCs, with age, sex, and TIV included as covariates. Threshold-free cluster enhancement (TFCE) was utilized to avoid arbitrary cluster-forming thresholds, and 5,000 permutations were performed to build an empirical null distribution. The statistical significance threshold was set at a family-wise error rate (FWER)-corrected *p*-value < 0.05.

Furthermore, to investigate the relationships among microstructural alterations, blood laboratory metrics, and cognitive performance, voxel-wise multiple linear regression analyses were conducted within the HTN group using FSL's randomize. SBP and MMSE scores were entered as variables of interest in separate models, with age, sex, and TIV controlled as covariates. Consistent with the group comparisons, TFCE was applied alongside 5,000 permutations, with significance defined as an FWER-corrected *p*-value < 0.05. To control the false-positive risk associated with conducting multiple voxel-wise tests across different diffusion metrics, FAt, MDt, FW, and the ALPS index were prespecified as our primary imaging outcomes, while conventional metrics were considered exploratory. Furthermore, to visualize the relationship between significant microstructural alterations and SBP, average metric values were extracted from the significant clusters and plotted. It is important to emphasize that these scatterplots are descriptive illustrations only; inferential statistics derived from these pre-selected clusters were not used as independent evidence to avoid circular analysis (double-dipping).

## 3 Results

### 3.1 Demographic characteristics

The study cohort comprised 60 participants, consisting of 30 patients diagnosed with HTN and 30 HCs. Detailed demographic, clinical, and laboratory profiles are summarized in Table 1. The two groups exhibited no statistically significant differences in age, sex distribution, or TIV.

**Table 1:** Demographic, clinical, and laboratory characteristics of the study cohort between the two groups [mean ± SD/median (Q1, Q3)].

Characteristics	HTN group	HC group	Effect size	95% CI	*t*/*z*/χ^2^ value	*p* value
Age (years)	58.00 (52.00, 61.00)	58.00 (51.00, 60.00)	0.030	[−2.500, 4.500]	0.230^b^	0.818
Sex (male/female)	M: 17, F: 13	M: 14, F: 16	0.100	[−0.152, 0.352]	0.601^c^	0.438
TIV (cm^3^)	1517.07 ± 109.10	1465.51 ± 134.85	0.420	[−11.836, 114.947]	1.628^a^	0.109
Duration (years)	6.98 ± 3.35	–	–	–	–	–
SBP (mmHg)	150.00 (139.70, 156.70)	125.10 (115.55, 130.32)	0.783	[16.500, 31.100]	6.012^b^	< 0.001
DBP (mmHg)	93.32 ± 11.53	77.37 ± 9.75	1.492	[10.375, 21.527]	5.729^a^	< 0.001
MAP (mmHg)	110.05 (103.90, 116.80)	91.20 (86.00, 96.70)	0.777	[11.350, 24.125]	5.816^b^	< 0.001
PP (mmHg)	59.29 ± 13.60	46.93 ± 10.59	1.004	[5.747, 18.965]	3.748^a^	< 0.001
MMSE (score)	28.00 (26.00, 30.00)	28.00 (26.00, 29.00)	0.054	[−1.500, 2.000]	0.422^b^	0.673
BMI (kg/m^2^)	25.75 ± 3.08	24.98 ± 3.50	0.235	[−0.930, 2.477]	0.909^a^	0.367
FBG (mmol/L)	5.01 ± 0.74	5.17 ± 0.80	0.202	[−0.558, 0.247]	−0.774^a^	0.442
HDL (mmol/L)	1.39 ± 0.25	1.35 ± 0.27	0.151	[−0.098, 0.178]	0.580^a^	0.564
LDL (mmol/L)	3.29 ± 1.04	3.21 ± 0.95	0.079	[−0.441, 0.597]	0.302^a^	0.763
TC (mmol/L)	5.21 ± 1.18	4.98 ± 0.77	0.225	[−0.297, 0.746]	0.863^a^	0.392
TG (mmol/L)	1.65 (1.24, 2.55)	1.55 (1.23, 1.89)	0.136	[−0.250, 0.750]	1.046^b^	0.295

Data are presented as mean ± standard deviation for normally distributed continuous variables, or median (interquartile range: Q1, Q3) for non-normally distributed data. Categorical variables are presented as counts. ^a^Indicates *t*-values obtained from the independent samples *t*-test. ^b^Indicates *z*-values obtained from the Mann–Whitney *U* test. ^c^Indicates χ^2^-values obtained from the Chi-square test. HTN, hypertension; HC, healthy control; CI, confidence interval; TIV, total intracranial volume; SBP, systolic blood pressure; DBP, diastolic blood pressure; MAP, mean arterial pressure; PP, pulse pressure; MMSE, mini-mental state examination; BMI, body mass index; FBG, fasting blood glucose; HDL, high-density lipoprotein; LDL, low-density lipoprotein; TC, total cholesterol; TG, triglycerides.

As anticipated for this clinical population, the HTN group demonstrated significantly elevated hemodynamic profiles across all measured indices compared to the HC group, including SBP, DBP, mean arterial pressure (MAP), and pulse pressure (PP) (all *p* < 0.001). Importantly, the cohorts showed no significant divergence in baseline global cognition, as assessed by the MMSE. Furthermore, basic anthropometric and metabolic panels, including body mass index (BMI), fasting blood glucose (FBG), high-density lipoprotein (HDL), low-density lipoprotein (LDL), total cholesterol (TC), and triglycerides (TG), remained comparable between the two groups. This rigorous baseline matching helps to minimize the influence of potential metabolic or demographic confounders, suggesting that subsequent microstructural differences are more likely associated with chronic hypertensive exposure.

### 3.2 Assessment of DTI-ALPS index

No statistically significant difference in the DTI-ALPS index was observed between the HTN group (mean ± SD: 1.62 ± 0.183) and the HC group [mean ± SD: 1.63 ± 0.175; adjusted mean difference: 0.013, 95% CI: (−0.076, 0.102), effect size (Cohen's *d*): 0.076, *t* = −0.295, *p* = 0.769]. Therefore, a group difference in macroscale glymphatic metrics could not be established in this sample.

### 3.3 TBSS results of WM

**Figure 1:**
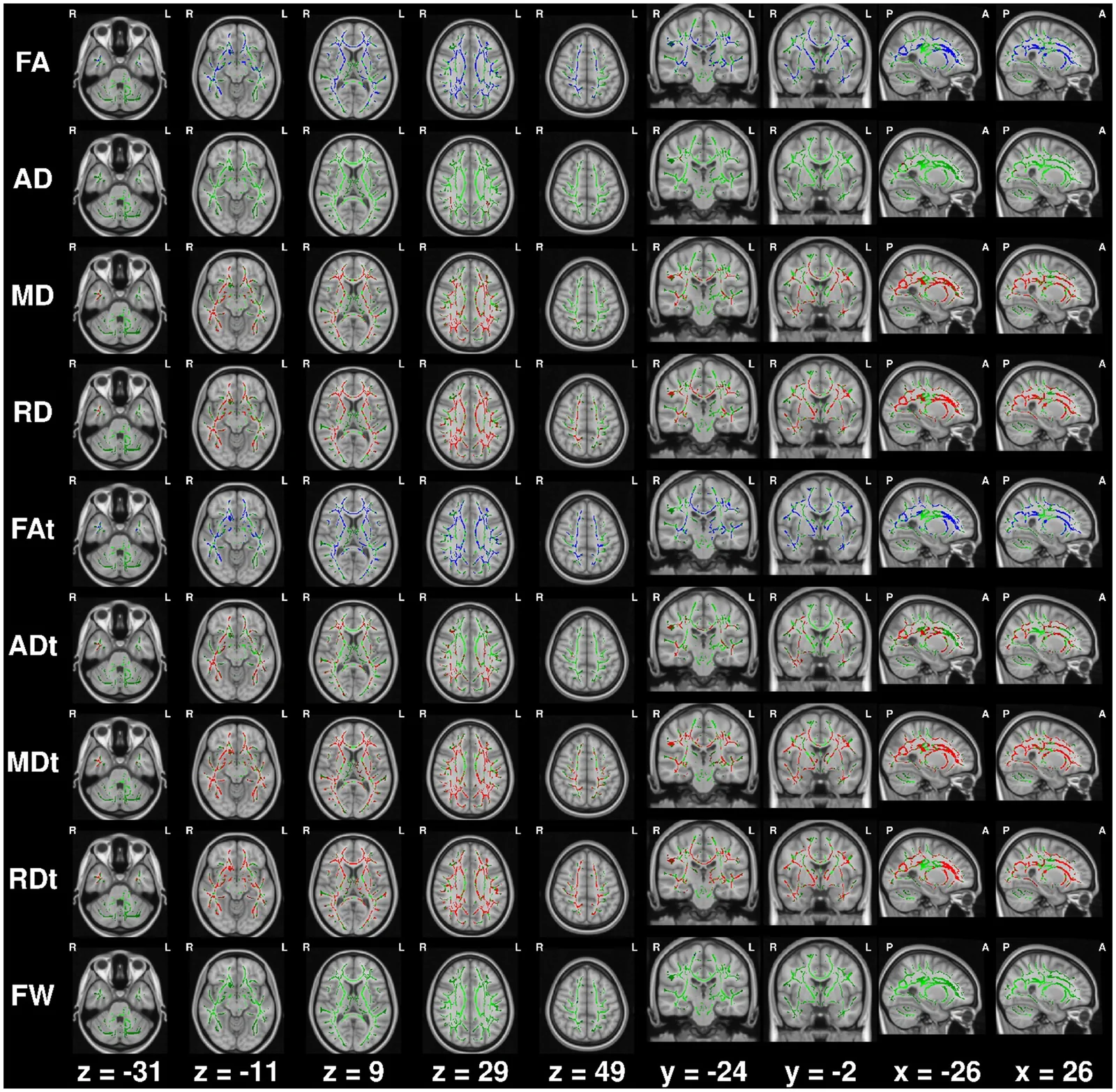
WM microstructural alterations in patients with HTN compared with HCs. The 9 × 9 matrix displays the spatial distribution of significant group differences across nine diffusion metrics. Rows from top to bottom represent the group differences in FA, AD, MD, RD, FAt, ADt, MDt, RDt, and FW, respectively. Columns from left to right display representative axial (*z* = −31, −11, 9, 29, 49), coronal (*y* = −24, −2), and sagittal (*x* = −26, 26) slices. Blue regions indicate significantly decreased values in the HTN group compared to the HC group, whereas red regions indicate significantly increased values. Statistical maps are thresholded at *p* < 0.05 (TFCE and FWER corrected) and overlaid on the standard MNI152 1-mm template. These color overlays represent binary significance masks rather than continuous statistical statistics. HTN, essential hypertension; HC, healthy control; FA, fractional anisotropy; AD, axial diffusivity; MD, mean diffusivity; RD, radial diffusivity; FAt, free-water-corrected FA; ADt, free-water-corrected AD; MDt, free-water-corrected MD; RDt, free-water-corrected RD; FW, fractional volume of free water; TFCE, threshold-free cluster enhancement; FWER, family-wise error rate.

Compared to HCs, patients with HTN exhibited widespread microstructural alterations across the WM skeleton (TFCE, FWER-corrected *p* < 0.05, Figure 1). In the conventional DTI analysis, the HTN group showed significantly decreased FA and increased MD, RD, and AD. These alterations were predominantly localized in the corpus callosum (genu, body, and splenium) and bilateral corona radiata, with MD and AD increases extending into the posterior thalamic radiations and superior longitudinal fasciculus.

Following FWE, the tissue-specific metrics demonstrated a spatial pattern largely consistent with the conventional metrics. Patients exhibited significantly reduced FAt and elevated MDt, RDt, and ADt, which were primarily concentrated in the corpus callosum and bilateral anterior corona radiata. Additionally, compared to HCs, the HTN group showed a significant decrease in the fractional volume of FW. The spatial extent of this reduced FW was highly localized, predominantly involving the body of the corpus callosum and the right superior corona radiata. Detailed information regarding cluster sizes, peak MNI coordinates, anatomical labels, and peak statistics for the WM skeleton are provided in Supplementary Table S1.

### 3.4 GBSS results

**Figure 2:**
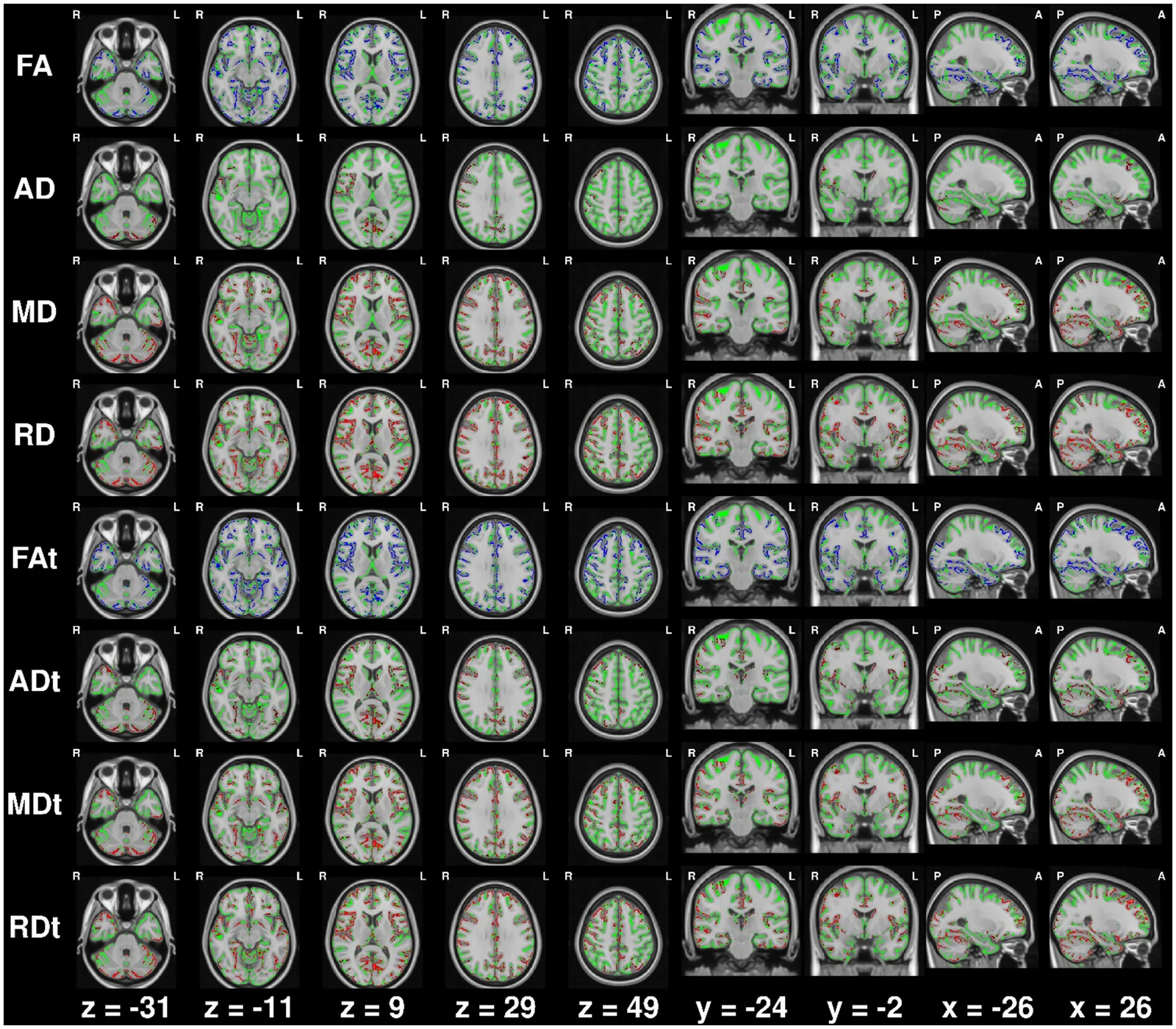
GM microstructural alterations in patients with HTN compared with HCs. The 8 × 9 matrix displays the spatial distribution of significant group differences across eight diffusion metrics. Rows from top to bottom represent the group differences in FA, AD, MD, RD, FAt, ADt, MDt, and RDt, respectively. Columns from left to right display representative axial (*z* = −31, −11, 9, 29, 49), coronal (*y* = −24, −2), and sagittal (*x* = −26, 26) slices. Blue regions indicate significantly decreased values in the HTN group compared to the HC group, whereas red regions indicate significantly increased values. Statistical maps are thresholded at *p* < 0.05 (TFCE and FWER corrected) and overlaid on the standard MNI152 1-mm template. These color overlays represent binary significance masks rather than continuous statistical statistics. GM, gray matter; HTN, essential hypertension; HC, healthy control; FA, fractional anisotropy; AD, axial diffusivity; MD, mean diffusivity; RD, radial diffusivity; FAt, free-water-corrected FA; ADt, free-water-corrected AD; MDt, free-water-corrected MD; RDt, free-water-corrected RD; TFCE, threshold-free cluster enhancement; FWER, family-wise error rate.

Compared to HCs, patients with HTN exhibited widespread microstructural alterations across the cortical GM skeleton (TFCE, FWER-corrected *p* < 0.05, Figure 2). In the conventional DTI analysis, the HTN group showed significantly decreased FA and increased MD, RD, and AD. These alterations were predominantly localized in the frontal cortical regions (including the frontal pole, superior frontal gyrus, and middle frontal gyrus), precentral and postcentral gyri, and insular cortex.

Following FWE, the tissue-specific metrics demonstrated a spatial pattern largely consistent with the conventional metrics. Patients exhibited significantly reduced FAt and elevated MDt, RDt, and ADt, which were primarily concentrated in similar cortical regions, prominently involving the frontal pole, precentral gyrus, and middle frontal gyrus. Detailed spatial coordinates, cluster sizes, and peak statistical values for the cortical GM skeleton are detailed in Supplementary Table S2.

### 3.5 Associations between microstructural metrics, blood pressure, and cognition

Within the HTN group, voxel-wise partial correlation analyses were performed to evaluate the relationships between diffusion metrics (in both WM and GM) and SBP, adjusting for age, sex, and TIV.

**Figure 3:**
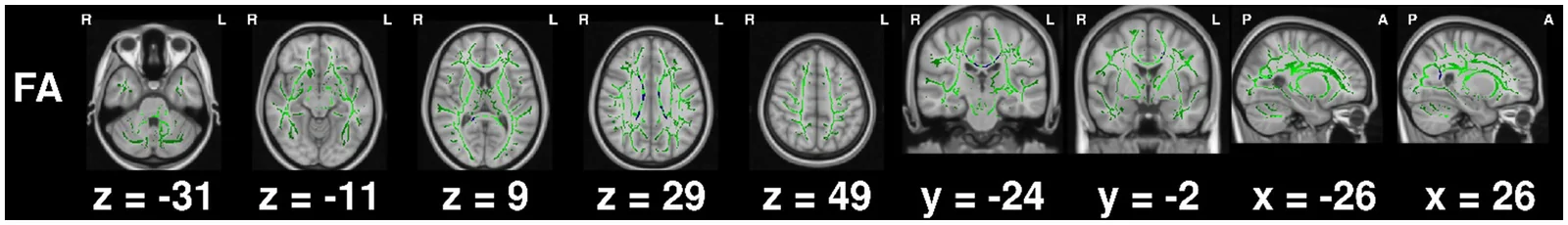
Voxel-wise TBSS correlation map between WM and SBP in patients with HTN. The brain map demonstrates significant negative associations between SBP and WM FA values, primarily located in the corpus callosum and corona radiata. Significant clusters (blue) are thresholded at *p* < 0.05 (TFCE and FWER corrected) and overlaid on the standard MNI152 1-mm template and the mean WM skeleton (green). These color overlays represent binary significance masks rather than continuous statistical statistics. WM, white matter; HTN, essential hypertension; SBP, systolic blood pressure; FA, fractional anisotropy; TBSS, tract-based spatial statistics; TFCE, threshold-free cluster enhancement; FWER, family-wise error rate.

**Figure 4:**
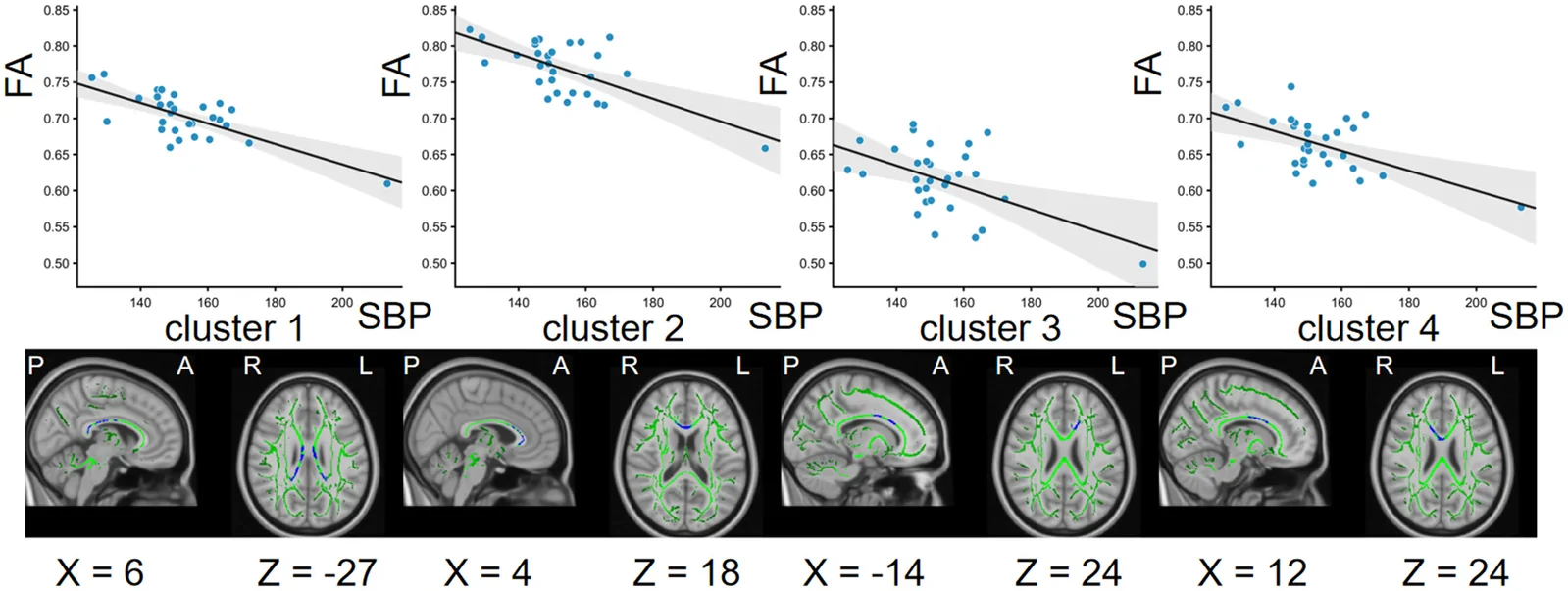
Descriptive scatter plots of the association between WM FA and SBP. The plots show covariate-adjusted residual FA values after adjustment for age, sex, and TIV in clusters identified in the same voxel-wise TBSS analysis. Because the clusters were selected from the same data, the plots are descriptive only and no additional inferential statistics are used as independent evidence. WM, white matter; SBP, systolic blood pressure; FA, fractional anisotropy; TIV, total intracranial volume; TBSS, tract-based spatial statistics.

In the WM skeleton, only conventional FA showed a significant negative association with SBP (TFCE, FWER-corrected *P* < 0.05; Figure 3). Significant clusters were located predominantly in the corpus callosum and bilateral corona radiata. Figure 4 displays covariate-adjusted residual FA values from these data-selected clusters for visualization only.

**Figure 5:**
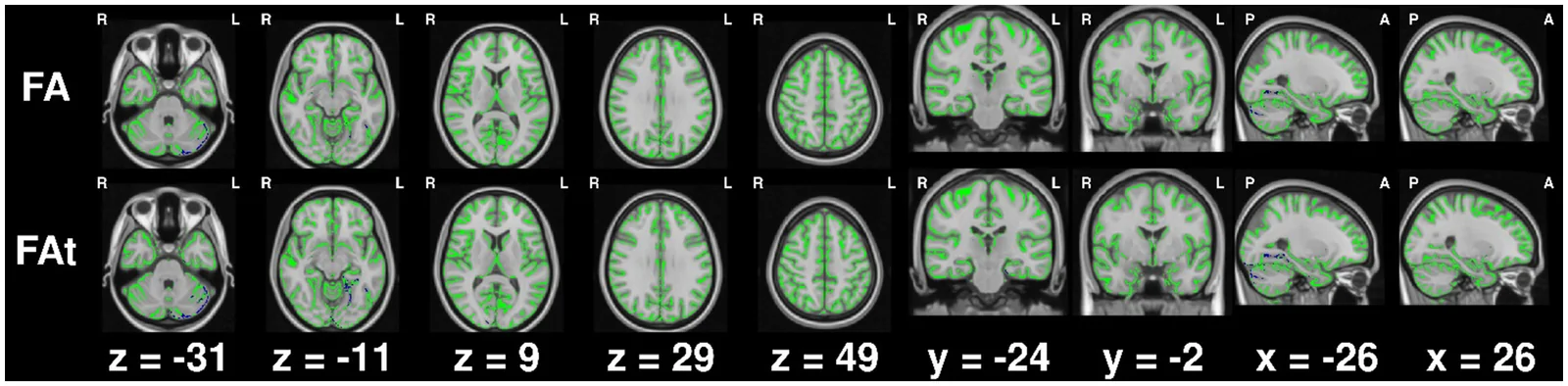
Voxel-wise GBSS correlation map between GM metrics and SBP in patients with HTN. The brain map demonstrates significant negative associations between SBP and cortical GM FA and FAt values. Significant clusters (blue) are thresholded at *p* < 0.05 (TFCE and FWER corrected) and overlaid on the standard MNI152 1-mm template and the mean GM skeleton (green). These color overlays represent binary significance masks rather than continuous statistical statistics. GM, gray matter; HTN, essential hypertension; SBP, systolic blood pressure; FA, fractional anisotropy; FAt, free-water-corrected FA; GBSS, GM-based spatial statistics; TFCE, threshold-free cluster enhancement; FWER, family-wise error rate.

**Figure 6:**
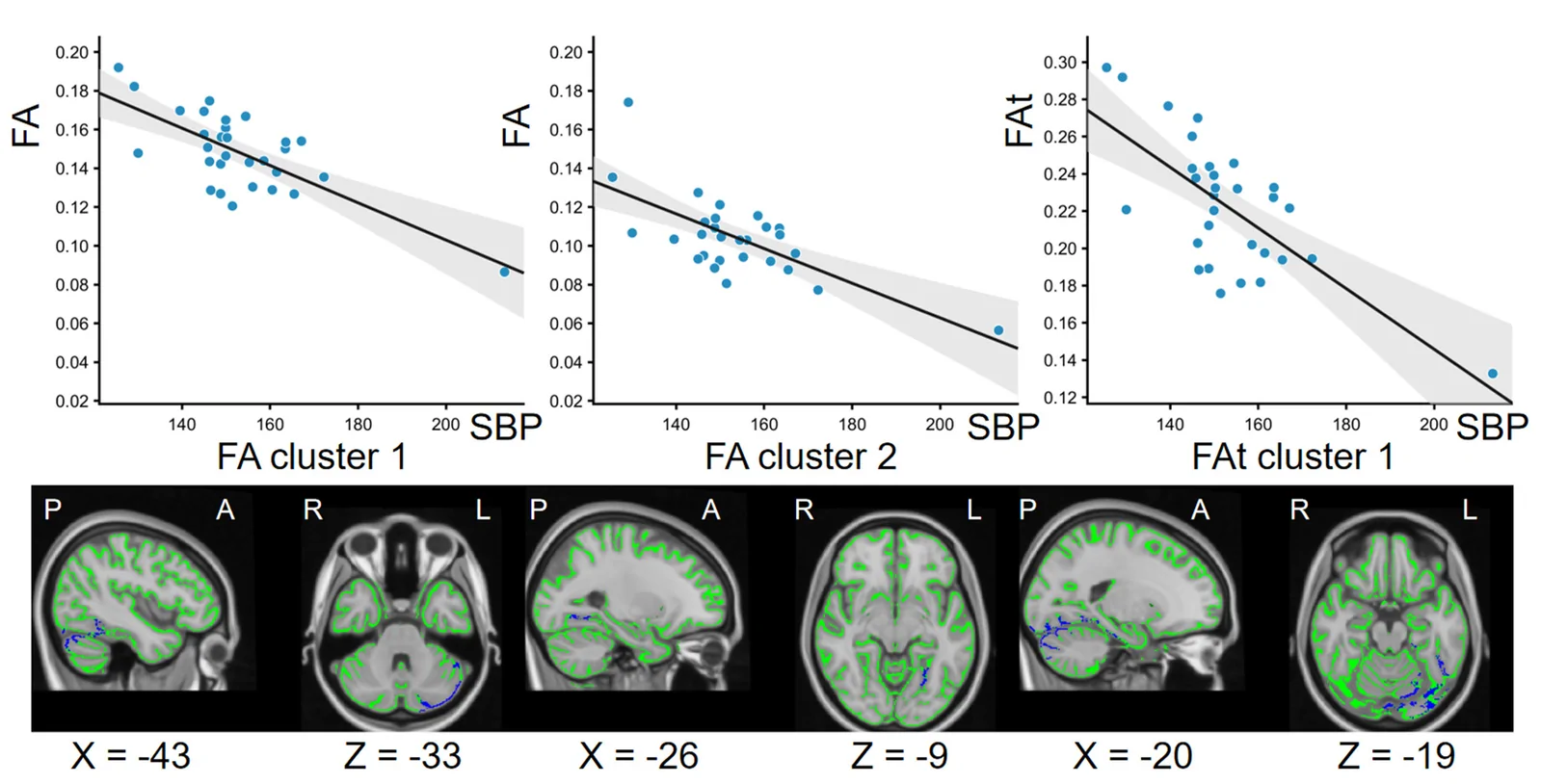
Descriptive scatter plots of the association between cortical GM diffusion metrics and SBP. The plots show covariate-adjusted residual values after adjustment for age, sex, and TIV in clusters identified in the same voxel-wise GBSS analysis. The first two columns show conventional FA clusters and the third column shows an FAt cluster. Because the clusters were selected from the same data, the plots are descriptive only and no additional inferential statistics are used as independent evidence. GM, gray matter; SBP, systolic blood pressure; FA, fractional anisotropy; FAt, free-water-corrected FA; TIV, total intracranial volume; GBSS, gray-matter-based spatial statistics.

In the cortical GM skeleton, conventional FA and FAt showed significant negative associations with SBP (TFCE, FWER-corrected *P* < 0.05; Figure 5). Figure 6 displays covariate-adjusted residual values from the same data-selected clusters for visualization only. Detailed cluster sizes, peak MNI coordinates, anatomical labels, and peak statistics for all significant WM and GM SBP associations are provided in Supplementary Table S3.

No significant correlations were observed between SBP and other diffusion metrics in either WM or GM. Additionally, no significant correlations were found between MMSE scores and any of the diffusion metrics across the whole-brain WM and GM skeletons in the HTN group.

## 4 Discussion

In this observational study, we investigated the interplay between microstructural alterations, extracellular fluid shifts, and macroscale glymphatic clearance in patients with essential HTN. Our findings point toward a potential difference in the onset of these pathological events: substantial tissue-specific microstructural abnormalities (reflected by reduced FAt and increased MDt) and localized reductions in FW were observed in the absence of significant macroscale glymphatic failure (evidenced by an unaltered DTI-ALPS index). This suggests that, in this specific clinical stage, the mechanical and ischemic burdens of elevated blood pressure may primarily affect the brain parenchyma and cellular microenvironment, while overt perivascular clearance pathways appear to remain functionally compensated. Consequently, by effectively isolating true tissue damage from extracellular fluid shifts, our results highlight the potential of FWE-derived metrics as sensitive neuroimaging indicators for observing subclinical hypertensive brain injury, possibly offering a window for observation without concurrent macroscopic glymphatic dysfunction or global cognitive declines manifest.

### 4.1 Absence of detectable ALPS differences and the sensitivity of FWE-derived metrics

The DTI-ALPS index is a recognized biomarker of glymphatic system efficiency, with reductions typically reported in advanced hypertension and established cerebral small vessel disease (34, 48). While some recent studies have also highlighted the potential of a reduced DTI-ALPS index as an early biomarker for preclinical cerebral small vessel disease and related cognitive vulnerability (35–37), the HTN group in our study showed no statistically significant reduction in the ALPS index relative to HCs. This observation implies that macroscopic perivascular clearance mechanisms may not have undergone measurable functional impairment in this specific cohort.

Importantly, despite the absence of ALPS abnormalities, our TBSS and GBSS analyses demonstrated widespread significant differences in FAt, MDt, and localized FW. This dissociation raises the possibility that tissue-specific microstructural alterations and localized fluid shifts could become detectable even before the emergence of measurable macroscale glymphatic dysfunction. Whereas, the ALPS index primarily reflects fluid movement along macroscopic perivascular spaces, FWE-DTI isolates the diffusion characteristics of the brain parenchyma and the cellular microenvironment (49). Therefore, the widespread reduction in FAt despite an unaltered ALPS index suggests that hypertensive mechanical stress may induce axonal vulnerability concurrently with, or possibly anticipating, the measurable impairment in perivascular clearance.

### 4.2 Potential tissue-specific microstructural alterations associated with HTN

Consistent with previous DTI studies (28, 50), we observed widespread reductions in FA and increases in diffusivity measures (MD, RD, and AD) in the HTN group. Importantly, our GBSS analysis extended these findings to the cortical GM. After the application of the FWE model, the tissue-specific metrics (FAt, MDt, RDt, and ADt) demonstrated a spatial pattern of abnormalities that was largely consistent with the conventional metrics. Rather than being redundant, this consistency is pathophysiologically highly informative. Conventional DTI indices are susceptible to partial volume effects from extracellular fluid (13, 14). In cortical GM specifically, a primary source of this effect is CSF contamination on the cortical surface, which causes a mathematical overestimation of diffusivity and an underestimation of FA (47). Consequently, metrics derived purely from conventional DTI models in GM often lack pathological sensitivity. By applying the FWE model, the concurrent widespread reduction in FAt rigorously indicates that the microstructural changes observed in our HTN cohort cannot be explained solely by extracellular fluid shifts or CSF contamination. Instead, these alterations likely reflect underlying microstructural vulnerabilities, such as myelin abnormalities, axonal alterations, or subtle cortical remodeling. Chronic elevation of blood pressure is known to promote microvascular remodeling and persistent hypoperfusion (51, 52), both of which may progressively increase regional axonal vulnerability.

### 4.3 Reduced free water as a possible indicator of early cellular stress

In contrast to the elevated FW commonly observed in severe atrophy, where tissue loss expands the extracellular space, our study demonstrated a significant reduction in FW within the corpus callosum and corona radiata in patients with HTN. This reduction in extracellular free water is a noteworthy finding (53). One speculative explanation for this localized FW reduction is subtle cellular swelling or reactive astrogliosis induced by chronic hypoxic-ischemic stress, rather than the classical cytotoxic edema typically associated with acute severe ischemia. Under conditions of mild but sustained hypoperfusion, disruption of ionic pump function may promote intracellular water influx, leading to cellular swelling and secondary compression of the extracellular space (54–56). The observation that this localized FW reduction was detectable while the ALPS index remained unaltered is more consistent with an early cellular stress response than with overt extracellular fluid stagnation. Accordingly, reduced FW in our cohort may represent a sensitive imaging marker of early neuroinflammatory or cellular stress-related changes in the hypertensive brain, before the development of gross perivascular clearance dysfunction.

### 4.4 Association between blood pressure and microstructural vulnerability

Our partial correlation analysis further supports a link between systemic hemodynamic burden and cerebral microstructural vulnerability. We found that SBP was extensively and negatively correlated with FA and FAt in both WM and GM skeletons. This relationship highlights the potential detrimental effect of elevated systolic pressure on brain tissue integrity. Increased systolic pressure may augment pulsatile energy transmission to the cerebral microvasculature, thereby potentially contributing to or exacerbating BBB disruption (6, 57). The particular vulnerability of the corpus callosum and frontal cortices further suggests that elevated SBP may adversely affect structural networks involved in higher-order cognitive processing (58, 59).

### 4.5 Absence of correlation with global cognitive scores

Despite the widespread tissue-specific microstructural abnormalities observed in HTN, we did not identify significant correlations between these imaging metrics and global cognitive performance as measured by the MMSE. This negative finding may partly reflect the limitations of the MMSE itself. As a global screening instrument developed primarily for dementia detection, the MMSE is known to exhibit ceiling effects and may lack sufficient sensitivity to detect subtle early impairments, especially domain-specific deficits in executive function and processing speed, which are considered early features of hypertensive brain injury (60, 61). As the MMSE has a pronounced ceiling effect in relatively high-functioning participants, we can only conclude that no association with global MMSE scores was detected in this sample. Further studies incorporating more sensitive, domain-specific neuropsychological batteries are required to determine the cognitive correlates of these microstructural changes.

### 4.6 Synthesis of pathophysiological mechanisms

Taken together, these findings support a preliminary pathophysiological model of early hypertensive brain involvement. At this stage of HTN, characterized by unaltered ALPS indices and intact global cognition on MMSE, the brain may already be undergoing substantial microstructural reorganization and cellular-level fluid redistribution. The mechanical and ischemic burden associated with elevated blood pressure may be linked to early cellular stress responses, potentially reflected by localized FW reduction, occurring alongside subtle intra-axonal and cortical abnormalities, as indicated by widespread changes in FAt and MDt. Importantly, the unaltered ALPS index and the lack of association with MMSE scores suggest that this cascade of microstructural and fluid alterations can be identified in the absence of overt macroscale glymphatic dysfunction or measurable global cognitive impairment. By helping to disentangle extracellular fluid shifts from tissue-specific abnormalities, the FWE model underscores the complex nature of brain vulnerability in HTN and highlights the need for sensitive imaging biomarkers capable of detecting preclinical hypertensive brain injury.

### 4.7 Limitations

Several limitations of this study should be acknowledged. First, the cross-sectional design precludes causal inference regarding the temporal relationship between elevated blood pressure and microstructural alterations. Second, the sample size is relatively small and drawn from a single center, requiring external validation in larger cohorts. Furthermore, the sample size was not based on a formal *a priori* power calculation, which may limit the statistical power to detect subtle group differences, such as in the DTI-ALPS index. Third, fitting a bi-tensor free-water model to single-shell data is mathematically underdetermined; although we utilized spatial regularization, the estimates remain sensitive to model assumptions. While recent advanced protocols utilize multi-shell acquisitions combined with non-linear least-squares approaches to further decrease free-water partial volume effects, our single-shell clinical data necessitates caution in interpreting the exact absolute values of the FW fraction. Fourth, the customized GBSS pipeline and the DTI-ALPS index, while carefully implemented, are indirect imaging proxies that can be influenced by local tissue geometry and registration procedures. Fifth, our statistical models adjusted for age, sex, and TIV, but did not account for several other potential clinical and demographic confounders. Specifically, detailed clinical data regarding specific antihypertensive medication classes, treatment adherence, and the long-term adequacy of blood pressure control were unavailable for this cohort. Furthermore, while factors such as education level, smoking and alcohol use, hypertension duration, and quantitative WMH volume are known modifiers of brain microstructure, they were not included as covariates in our voxel-wise analyses. Given our relatively small sample size, introducing numerous additional covariates would risk overfitting the models and severely compromising statistical power. Although severe macroscopic lesions were excluded, residual confounding from mild cerebral small vessel disease and these unmeasured clinical variables cannot be entirely ruled out. Furthermore, our study has methodological limitations regarding the DTI-ALPS analysis, which is an indirect diffusion-based proxy, its calculation depends heavily on local tissue geometry. Therefore, the ALPS measurements in our study might be partially influenced by the concurrent widespread white matter microstructural abnormalities we observed in the hypertensive cohort. Finally, addressing the multiplicity of statistical testing across numerous imaging metrics inherently increases the risk of false positives, necessitating caution in interpreting the exploratory metric associations.

## 5 Conclusion

In conclusion, by combining a single-shell FWE-DTI approach with ALPS analysis, this study demonstrates that tissue-specific microstructural abnormalities and localized reductions in extracellular free water are detectable in HTN, even when macroscale glymphatic clearance, as measured by the ALPS index, does not show statistically significant impairment. The widespread reductions in FAt, together with localized FW decreases, provide novel insight into the neuropathological processes of HTN and suggest potential contributions of cellular stress and microstructural vulnerability at a stage when overt glymphatic dysfunction is not yet clinically apparent. Moreover, the negative associations between systolic blood pressure and tissue-specific microstructural integrity generate the hypothesis that early and rigorous blood pressure control might mitigate the progression of these subclinical alterations. This hypothesis remains to be tested in future longitudinal interventional studies.

## Data Availability

The raw data supporting the conclusions of this article will be made available by the authors, without undue reservation.
