# Supplementary material for: Tissue-specific diffusion abnormalities and localized free-water reductions in hypertension despite no detectable group difference in the DTI-ALPS index
Source: Front Med (Lausanne). 2026 Aug 12;13:1867342. doi: 10.3389/fmed.2026.1867342 (PMC13506801; doi:10.3389/fmed.2026.1867342)
Supplement: Supplementary file 1 [file Table_1.docx]

**Fig S1. Participant-flow diagram of the study cohort.** The diagram illustrates the sequential process of screening, exclusions, and final cohort selection. A total of 60 potential participants were screened for eligibility at the 910th Hospital of the PLA Joint Logistics Support Force between July 2016 and December 2025. Following clinical exclusion criteria, including a history of major neurological/psychiatric disorders, specific systemic diseases, and the use of cognition-affecting medications, eligible participants underwent structural and diffusion MRI. Subsequent exclusions were based on imaging findings (e.g., macroscopic intracranial pathology or visually severe, confluent white matter lesions) and MRI QC failure. The final analytical cohort comprised 60 participants, equally divided into patients with HTN (n = 30) and HCs (n = 30). These participants were included in the downstream DTI-ALPS, TBSS, GBSS, and voxel-wise analyses. PLA, People's Liberation Army; MRI, magnetic resonance imaging; QC, quality control; HTN, essential hypertension; HC, healthy control; DTI-ALPS, diffusion tensor imaging analysis along the perivascular space; TBSS, tract-based spatial statistics; GBSS, gray matter-based spatial statistics; MMSE, Mini-Mental State Examination.
