# Supplementary material for: Tissue-specific diffusion abnormalities and localized free-water reductions in hypertension despite no detectable group difference in the DTI-ALPS index
Source: Front Med (Lausanne). 2026 Aug 12;13:1867342. doi: 10.3389/fmed.2026.1867342 (PMC13506801; doi:10.3389/fmed.2026.1867342)
Supplement: Supplementary file 2 [file Table_2.docx]

**Fig. S2. Representative native-space free-water fraction map.** Representative native-space free-water fraction (FWF) maps for one control participant. The top row shows the mean b=0 image; the bottom row shows the corresponding FWF maps within the participant-specific brain mask. The scale ranges from 0 to 1; higher values (yellow-to-red) are expected in CSF-rich spaces, including ventricular and sulcal regions. Images are displayed using the radiological convention (R on image left, L on image right).
