# Supplementary material for: Tissue-specific diffusion abnormalities and localized free-water reductions in hypertension despite no detectable group difference in the DTI-ALPS index
Source: Front Med (Lausanne). 2026 Aug 12;13:1867342. doi: 10.3389/fmed.2026.1867342 (PMC13506801; doi:10.3389/fmed.2026.1867342)
Supplement: Supplementary file 3 [file Table_3.docx]

**Supplementary Figure S3: Individual FA Skeleton Projection Quality Control: TBSS and GBSS**

Red: individual subject's skeleton-projected FA. Radiological display: R is on the image left; L is on the image right.

**TBSS**

|   Sub 1 |   Sub 2 |   Sub 3 |   Sub 4 |   Sub 5 |   Sub 6 |   Sub 7 |   Sub 8 |   Sub 9 |   Sub 10 |
| --- | --- | --- | --- | --- | --- | --- | --- | --- | --- |
|   Sub 11 |   Sub 12 |   Sub 13 |   Sub 14 |   Sub 15 |   Sub 16 |   Sub 17 |   Sub 18 |   Sub 19 |   Sub 20 |
|   Sub 21 |   Sub 22 |   Sub 23 |   Sub 24 |   Sub 25 |   Sub 26 |   Sub 27 |   Sub 28 |   Sub 29 |   Sub 30 |
|   Sub 31 |   Sub 32 |   Sub 33 |   Sub 34 |   Sub 35 |   Sub 36 |   Sub 37 |   Sub 38 |   Sub 39 |   Sub 40 |
|   Sub 41 |   Sub 42 |   Sub 43 |   Sub 44 |   Sub 45 |   Sub 46 |   Sub 47 |   Sub 48 |   Sub 49 |   Sub 50 |
|   Sub 51 |   Sub 52 |   Sub 53 |   Sub 54 |   Sub 55 |   Sub 56 |   Sub 57 |   Sub 58 |   Sub 59 |   Sub 60 |

R	L

**GBSS**

|   Sub 1 |   Sub 2 |   Sub 3 |   Sub 4 |   Sub 5 |   Sub 6 |   Sub 7 |   Sub 8 |   Sub 9 |   Sub 10 |
| --- | --- | --- | --- | --- | --- | --- | --- | --- | --- |
|   Sub 11 |   Sub 12 |   Sub 13 |   Sub 14 |   Sub 15 |   Sub 16 |   Sub 17 |   Sub 18 |   Sub 19 |   Sub 20 |
|   Sub 21 |   Sub 22 |   Sub 23 |   Sub 24 |   Sub 25 |   Sub 26 |   Sub 27 |   Sub 28 |   Sub 29 |   Sub 30 |
|   Sub 31 |   Sub 32 |   Sub 33 |   Sub 34 |   Sub 35 |   Sub 36 |   Sub 37 |   Sub 38 |   Sub 39 |   Sub 40 |
| Sub 41 | Sub 42 | Sub 43 | Sub 44 | Sub 45 | Sub 46 | Sub 47 | Sub 48 | Sub 49 | Sub 50 |
| Sub 51 | Sub 52 | Sub 53 | Sub 54 | Sub 55 | Sub 56 | Sub 57 | Sub 58 | Sub 59 | Sub 60 |

R	L
