# Supplementary material for: Tissue-specific diffusion abnormalities and localized free-water reductions in hypertension despite no detectable group difference in the DTI-ALPS index
Source: Front Med (Lausanne). 2026 Aug 12;13:1867342. doi: 10.3389/fmed.2026.1867342 (PMC13506801; doi:10.3389/fmed.2026.1867342)
Supplement: Supplementary file 4 [file Table_4.docx]

**Supplementary Tables: Voxel-Wise Diffusion MRI Results**

**Table S1. White-matter TBSS group-comparison clusters**

*Direction and descriptives refer to HTN relative to HC. All listed clusters survived TFCE FWER correction at P < 0.05.*

| Metric | Cluster (voxels) | Peak MNI (x, y, z) | Anatomical labels | Direction | HTN mean ± SD | HC mean ± SD | HTN-HC 95% CI | Hedges g | Peak statistic | Min FWER P |
| --- | --- | --- | --- | --- | --- | --- | --- | --- | --- | --- |
| FA | 47742 | (22, 10, 23) | Body of corpus callosum; Anterior corona radiata L; Genu of corpus callosum | HTN < HC | 0.5048 ± 0.0211 | 0.5357 ± 0.0167 | -0.0309 (-0.0406, -0.0213) | -1.603 | 6.583 | 0.0004 |
| MD | 45736 | (22, 21, -8) | Body of corpus callosum; Anterior corona radiata L; Splenium of corpus callosum | HTN > HC | 0.0008 ± 0.0000 | 0.0007 ± 0.0000 | 0.0000 (0.0000, 0.0001) | 1.451 | 5.826 | 0.0018 |
| AD | 886 | (-35, -47, 28) | Posterior thalamic radiation (include optic radiation) L; Splenium of corpus callosum; Superior longitudinal fasciculus L | HTN > HC | 0.0013 ± 0.0000 | 0.0013 ± 0.0000 | 0.0001 (0.0000, 0.0001) | 1.453 | 4.309 | 0.0324 |
| AD | 1225 | (35, -44, 31) | Superior longitudinal fasciculus R; Posterior thalamic radiation (include optic radiation) R; Posterior corona radiata R | HTN > HC | 0.0013 ± 0.0001 | 0.0012 ± 0.0000 | 0.0001 (0.0000, 0.0001) | 1.442 | 4.835 | 0.0220 |
| RD | 53812 | (22, 9, 23) | Body of corpus callosum; Anterior corona radiata L; Splenium of corpus callosum | HTN > HC | 0.0005 ± 0.0000 | 0.0005 ± 0.0000 | 0.0000 (0.0000, 0.0001) | 1.446 | 6.366 | 0.0006 |
| FAt | 38 | (31, 20, 39) | Unclassified | HTN < HC | 0.7684 ± 0.0791 | 0.8379 ± 0.0627 | -0.0695 (-0.1056, -0.0334) | -0.961 | 4.839 | 0.0476 |
| FAt | 51634 | (22, 10, 23) | Body of corpus callosum; Anterior corona radiata L; Genu of corpus callosum | HTN < HC | 0.7639 ± 0.0267 | 0.8010 ± 0.0153 | -0.0371 (-0.0481, -0.0261) | -1.682 | 6.910 | <0.0002 |
| MDt | 64760 | (22, 21, -8) | Anterior corona radiata L; Body of corpus callosum; Splenium of corpus callosum | HTN > HC | 0.0004 ± 0.0000 | 0.0004 ± 0.0000 | 0.0000 (0.0000, 0.0000) | 1.396 | 6.475 | 0.0012 |
| ADt | 14746 | (-24, -54, 15) | Splenium of corpus callosum; Anterior corona radiata L; Superior corona radiata L | HTN > HC | 0.0009 ± 0.0000 | 0.0009 ± 0.0000 | 0.0000 (0.0000, 0.0001) | 1.799 | 5.127 | 0.0050 |
| ADt | 16608 | (30, 24, 33) | Anterior corona radiata R; Superior longitudinal fasciculus R; Superior corona radiata R | HTN > HC | 0.0009 ± 0.0000 | 0.0008 ± 0.0000 | 0.0001 (0.0000, 0.0001) | 1.538 | 5.471 | 0.0036 |
| RDt | 63032 | (22, 9, 23) | Body of corpus callosum; Anterior corona radiata L; Splenium of corpus callosum | HTN > HC | 0.0002 ± 0.0000 | 0.0002 ± 0.0000 | 0.0000 (0.0000, 0.0000) | 1.528 | 7.249 | 0.0004 |
| FW | 2 | (11, -37, 60) | Unclassified | HTN < HC | 0.2778 ± 0.0281 | 0.3126 ± 0.0314 | -0.0348 (-0.0499, -0.0197) | -1.153 | 4.467 | 0.0500 |
| FW | 6 | (-12, -24, 64) | Unclassified | HTN < HC | 0.3204 ± 0.0220 | 0.3467 ± 0.0251 | -0.0263 (-0.0382, -0.0143) | -1.099 | 4.217 | 0.0500 |
| FW | 58 | (-9, -34, 63) | Unclassified | HTN < HC | 0.2783 ± 0.0275 | 0.3070 ± 0.0141 | -0.0287 (-0.0398, -0.0176) | -1.295 | 4.677 | 0.0500 |
| FW | 379 | (13, -25, 61) | Body of corpus callosum; Superior corona radiata R | HTN < HC | 0.3287 ± 0.0131 | 0.3479 ± 0.0105 | -0.0192 (-0.0252, -0.0132) | -1.596 | 4.158 | 0.0440 |

*Abbreviations: FA, fractional anisotropy; FAt, free-water-corrected FA; MD, mean diffusivity; MDt, free-water-corrected MD; AD/RD, axial/radial diffusivity; ADt/RDt, free-water-corrected axial/radial diffusivity; FW, free-water fractional volume.*

**Table S2. Cortical gray-matter GBSS group-comparison clusters**

*Direction and descriptives refer to HTN relative to HC. All listed clusters survived TFCE FWER correction at P < 0.05.*

| Metric | Cluster (voxels) | Peak MNI (x, y, z) | Anatomical labels | Direction | HTN mean ± SD | HC mean ± SD | HTN-HC 95% CI | Hedges g | Peak statistic | Min FWER P |
| --- | --- | --- | --- | --- | --- | --- | --- | --- | --- | --- |
| FA | 93177 | (40, -90, -11) | Frontal Pole; Middle Frontal Gyrus; Insular Cortex | HTN < HC | 0.1219 ± 0.0080 | 0.1427 ± 0.0100 | -0.0208 (-0.0254, -0.0162) | -2.267 | 5.572 | 0.0008 |
| MD | 1 | (-46, -41, 46) | Supramarginal Gyrus, posterior division; Supramarginal Gyrus, anterior division; Superior Parietal Lobule | HTN > HC | 0.0013 ± 0.0002 | 0.0011 ± 0.0002 | 0.0002 (0.0001, 0.0003) | 0.868 | 3.345 | 0.0500 |
| MD | 53 | (-40, -39, 43) | Postcentral Gyrus; Superior Parietal Lobule; Supramarginal Gyrus, anterior division | HTN > HC | 0.0013 ± 0.0002 | 0.0011 ± 0.0002 | 0.0002 (0.0001, 0.0003) | 1.044 | 3.705 | 0.0492 |
| MD | 114 | (-50, -37, 46) | Supramarginal Gyrus, anterior division; Postcentral Gyrus; Superior Parietal Lobule | HTN > HC | 0.0013 ± 0.0002 | 0.0011 ± 0.0002 | 0.0002 (0.0001, 0.0003) | 1.076 | 5.235 | 0.0456 |
| MD | 94750 | (-23, 5, -15) | Frontal Pole; Lateral Occipital Cortex, superior division; Precuneous Cortex | HTN > HC | 0.0010 ± 0.0001 | 0.0009 ± 0.0001 | 0.0001 (0.0001, 0.0001) | 2.087 | 6.202 | 0.0042 |
| AD | 23 | (-2, -90, 28) | Occipital Pole; Cuneal Cortex | HTN > HC | 0.0013 ± 0.0003 | 0.0010 ± 0.0003 | 0.0003 (0.0001, 0.0004) | 0.954 | 3.984 | 0.0496 |
| AD | 24 | (33, 0, -5) | Insular Cortex | HTN > HC | 0.0013 ± 0.0001 | 0.0011 ± 0.0001 | 0.0001 (0.0001, 0.0002) | 1.028 | 3.772 | 0.0492 |
| AD | 28 | (-2, 34, -25) | Frontal Medial Cortex; Subcallosal Cortex; Frontal Pole | HTN > HC | 0.0013 ± 0.0002 | 0.0010 ± 0.0003 | 0.0002 (0.0001, 0.0004) | 0.984 | 3.973 | 0.0496 |
| AD | 118 | (-33, -87, 7) | Lateral Occipital Cortex, superior division; Lateral Occipital Cortex, inferior division; Occipital Pole | HTN > HC | 0.0011 ± 0.0001 | 0.0010 ± 0.0001 | 0.0001 (0.0001, 0.0002) | 1.037 | 3.609 | 0.0484 |
| AD | 140 | (5, -55, 36) | Precuneous Cortex; Cingulate Gyrus, posterior division; Superior Parietal Lobule | HTN > HC | 0.0011 ± 0.0001 | 0.0010 ± 0.0001 | 0.0001 (0.0000, 0.0001) | 0.793 | 3.113 | 0.0492 |
| AD | 270 | (43, 21, -34) | Temporal Pole; Temporal Fusiform Cortex, anterior division; Inferior Temporal Gyrus, anterior division | HTN > HC | 0.0011 ± 0.0001 | 0.0009 ± 0.0001 | 0.0002 (0.0001, 0.0002) | 1.314 | 4.271 | 0.0448 |
| AD | 283 | (11, 56, 36) | Frontal Pole; Superior Frontal Gyrus | HTN > HC | 0.0012 ± 0.0002 | 0.0010 ± 0.0001 | 0.0001 (0.0001, 0.0002) | 0.993 | 4.151 | 0.0428 |
| AD | 469 | (-11, 1, 16) | Unclassified | HTN > HC | 0.0014 ± 0.0001 | 0.0012 ± 0.0001 | 0.0002 (0.0001, 0.0003) | 1.359 | 5.330 | 0.0366 |
| AD | 837 | (41, 27, -18) | Frontal Orbital Cortex; Frontal Pole; Subcallosal Cortex | HTN > HC | 0.0011 ± 0.0001 | 0.0010 ± 0.0001 | 0.0001 (0.0001, 0.0002) | 1.208 | 3.551 | 0.0466 |
| AD | 5962 | (20, -64, 18) | Precuneous Cortex; Intracalcarine Cortex; Cingulate Gyrus, posterior division | HTN > HC | 0.0013 ± 0.0001 | 0.0012 ± 0.0001 | 0.0001 (0.0001, 0.0001) | 1.251 | 4.511 | 0.0240 |
| AD | 8133 | (-48, -59, -28) | Occipital Fusiform Gyrus; Occipital Pole; Lingual Gyrus | HTN > HC | 0.0011 ± 0.0001 | 0.0010 ± 0.0002 | 0.0001 (0.0001, 0.0002) | 0.924 | 3.924 | 0.0290 |
| AD | 9176 | (59, 21, 9) | Middle Frontal Gyrus; Precentral Gyrus; Insular Cortex | HTN > HC | 0.0013 ± 0.0001 | 0.0011 ± 0.0001 | 0.0001 (0.0001, 0.0002) | 1.678 | 4.996 | 0.0230 |
| RD | 113951 | (29, 31, 49) | Frontal Pole; Lateral Occipital Cortex, superior division; Precuneous Cortex | HTN > HC | 0.0010 ± 0.0001 | 0.0009 ± 0.0001 | 0.0001 (0.0001, 0.0001) | 1.902 | 5.198 | 0.0032 |
| FAt | 7 | (-10, -74, 53) | Lateral Occipital Cortex, superior division; Precuneous Cortex | HTN < HC | 0.1540 ± 0.0667 | 0.1830 ± 0.0723 | -0.0290 (-0.0642, 0.0062) | -0.411 | 1.940 | 0.0500 |
| FAt | 14 | (-5, -72, 56) | Precuneous Cortex; Lateral Occipital Cortex, superior division | HTN < HC | 0.1649 ± 0.0475 | 0.1956 ± 0.0697 | -0.0307 (-0.0609, -0.0005) | -0.509 | 2.482 | 0.0500 |
| FAt | 19 | (-9, -75, 56) | Lateral Occipital Cortex, superior division; Precuneous Cortex | HTN < HC | 0.1401 ± 0.0478 | 0.1681 ± 0.0747 | -0.0281 (-0.0598, 0.0037) | -0.442 | 2.093 | 0.0500 |
| FAt | 102 | (23, -45, 59) | Superior Parietal Lobule; Postcentral Gyrus; Lateral Occipital Cortex, superior division | HTN < HC | 0.1829 ± 0.0636 | 0.2409 ± 0.0883 | -0.0581 (-0.0970, -0.0191) | -0.745 | 2.910 | 0.0482 |
| FAt | 109 | (-14, -64, 60) | Lateral Occipital Cortex, superior division; Precuneous Cortex; Superior Parietal Lobule | HTN < HC | 0.1791 ± 0.0454 | 0.2201 ± 0.0769 | -0.0410 (-0.0729, -0.0090) | -0.640 | 2.818 | 0.0496 |
| FAt | 111139 | (61, -57, -15) | Frontal Pole; Lateral Occipital Cortex, superior division; Middle Frontal Gyrus | HTN < HC | 0.2061 ± 0.0165 | 0.2442 ± 0.0187 | -0.0381 (-0.0470, -0.0291) | -2.127 | 5.615 | 0.0004 |
| MDt | 2 | (48, 3, -40) | Inferior Temporal Gyrus, anterior division; Temporal Pole; Middle Temporal Gyrus, anterior division | HTN > HC | 0.0005 ± 0.0001 | 0.0004 ± 0.0002 | 0.0001 (0.0000, 0.0002) | 0.721 | 2.755 | 0.0500 |
| MDt | 3 | (46, 2, -39) | Inferior Temporal Gyrus, anterior division; Temporal Pole; Middle Temporal Gyrus, anterior division | HTN > HC | 0.0005 ± 0.0001 | 0.0004 ± 0.0001 | 0.0001 (0.0000, 0.0001) | 0.679 | 2.908 | 0.0500 |
| MDt | 6 | (-30, -33, -19) | Temporal Fusiform Cortex, posterior division; Parahippocampal Gyrus, posterior division; Temporal Occipital Fusiform Cortex | HTN > HC | 0.0005 ± 0.0001 | 0.0005 ± 0.0001 | 0.0001 (0.0000, 0.0001) | 0.853 | 2.831 | 0.0500 |
| MDt | 24 | (-31, -41, -13) | Temporal Fusiform Cortex, posterior division; Parahippocampal Gyrus, posterior division; Temporal Occipital Fusiform Cortex | HTN > HC | 0.0006 ± 0.0001 | 0.0005 ± 0.0001 | 0.0001 (0.0000, 0.0001) | 0.716 | 2.637 | 0.0500 |
| MDt | 24 | (-40, -32, -23) | Temporal Fusiform Cortex, posterior division; Inferior Temporal Gyrus, posterior division | HTN > HC | 0.0006 ± 0.0001 | 0.0005 ± 0.0001 | 0.0001 (0.0000, 0.0001) | 0.844 | 3.245 | 0.0496 |
| MDt | 25 | (47, 0, -34) | Inferior Temporal Gyrus, anterior division; Middle Temporal Gyrus, anterior division; Inferior Temporal Gyrus, posterior division | HTN > HC | 0.0005 ± 0.0001 | 0.0005 ± 0.0001 | 0.0001 (0.0000, 0.0001) | 0.832 | 2.988 | 0.0494 |
| MDt | 34 | (-46, -38, 25) | Parietal Operculum Cortex; Planum Temporale; Supramarginal Gyrus, posterior division | HTN > HC | 0.0008 ± 0.0003 | 0.0007 ± 0.0002 | 0.0001 (0.0000, 0.0002) | 0.642 | 2.818 | 0.0496 |
| MDt | 112 | (-57, -43, 27) | Supramarginal Gyrus, posterior division; Parietal Operculum Cortex; Supramarginal Gyrus, anterior division | HTN > HC | 0.0009 ± 0.0003 | 0.0007 ± 0.0002 | 0.0002 (0.0000, 0.0003) | 0.718 | 2.873 | 0.0490 |
| MDt | 3430 | (35, -93, 10) | Lateral Occipital Cortex, inferior division; Occipital Pole; Lateral Occipital Cortex, superior division | HTN > HC | 0.0008 ± 0.0001 | 0.0007 ± 0.0001 | 0.0001 (0.0001, 0.0001) | 1.484 | 4.577 | 0.0332 |
| MDt | 87925 | (-21, -54, -18) | Frontal Pole; Precuneous Cortex; Middle Frontal Gyrus | HTN > HC | 0.0008 ± 0.0001 | 0.0007 ± 0.0000 | 0.0001 (0.0001, 0.0001) | 1.977 | 5.371 | 0.0062 |
| ADt | 16 | (10, 57, -25) | Frontal Pole; Frontal Medial Cortex | HTN > HC | 0.0005 ± 0.0004 | 0.0003 ± 0.0003 | 0.0002 (0.0000, 0.0004) | 0.626 | 2.269 | 0.0498 |
| ADt | 17 | (-36, 33, -11) | Frontal Orbital Cortex; Frontal Pole | HTN > HC | 0.0008 ± 0.0001 | 0.0007 ± 0.0001 | 0.0001 (0.0000, 0.0002) | 0.783 | 3.179 | 0.0494 |
| ADt | 21 | (-34, 30, -20) | Frontal Orbital Cortex; Frontal Pole | HTN > HC | 0.0009 ± 0.0002 | 0.0007 ± 0.0002 | 0.0001 (0.0000, 0.0002) | 0.660 | 2.711 | 0.0498 |
| ADt | 22 | (-43, 49, 16) | Frontal Pole | HTN > HC | 0.0009 ± 0.0003 | 0.0007 ± 0.0001 | 0.0002 (0.0001, 0.0003) | 0.786 | 2.579 | 0.0494 |
| ADt | 27 | (53, -25, -5) | Middle Temporal Gyrus, posterior division; Superior Temporal Gyrus, posterior division; Supramarginal Gyrus, posterior division | HTN > HC | 0.0008 ± 0.0002 | 0.0007 ± 0.0001 | 0.0001 (0.0000, 0.0002) | 0.823 | 2.961 | 0.0492 |
| ADt | 39 | (-4, 10, 26) | Cingulate Gyrus, anterior division | HTN > HC | 0.0009 ± 0.0001 | 0.0007 ± 0.0001 | 0.0001 (0.0001, 0.0002) | 1.079 | 3.877 | 0.0490 |
| ADt | 42 | (60, -21, -6) | Middle Temporal Gyrus, posterior division; Superior Temporal Gyrus, posterior division | HTN > HC | 0.0009 ± 0.0002 | 0.0008 ± 0.0002 | 0.0001 (0.0000, 0.0002) | 0.684 | 2.610 | 0.0492 |
| ADt | 44 | (4, 61, -3) | Frontal Pole; Frontal Medial Cortex; Paracingulate Gyrus | HTN > HC | 0.0011 ± 0.0003 | 0.0008 ± 0.0002 | 0.0003 (0.0002, 0.0005) | 1.148 | 4.381 | 0.0476 |
| ADt | 55 | (8, 51, -21) | Frontal Pole; Frontal Medial Cortex | HTN > HC | 0.0006 ± 0.0003 | 0.0004 ± 0.0003 | 0.0002 (0.0001, 0.0003) | 0.724 | 3.403 | 0.0490 |
| ADt | 85 | (-27, 31, -14) | Frontal Orbital Cortex; Frontal Pole | HTN > HC | 0.0008 ± 0.0001 | 0.0007 ± 0.0001 | 0.0001 (0.0001, 0.0001) | 1.221 | 2.999 | 0.0492 |
| ADt | 105 | (-2, 6, 35) | Cingulate Gyrus, anterior division; Juxtapositional Lobule Cortex (formerly Supplementary Motor Cortex); Paracingulate Gyrus | HTN > HC | 0.0009 ± 0.0001 | 0.0008 ± 0.0001 | 0.0001 (0.0001, 0.0002) | 0.982 | 3.786 | 0.0490 |
| ADt | 110 | (-52, 39, 2) | Frontal Pole; Inferior Frontal Gyrus, pars triangularis; Frontal Orbital Cortex | HTN > HC | 0.0009 ± 0.0001 | 0.0007 ± 0.0001 | 0.0002 (0.0001, 0.0002) | 1.265 | 3.847 | 0.0478 |
| ADt | 195 | (6, 51, -12) | Frontal Medial Cortex; Paracingulate Gyrus; Frontal Pole | HTN > HC | 0.0009 ± 0.0002 | 0.0008 ± 0.0001 | 0.0002 (0.0001, 0.0002) | 1.238 | 4.450 | 0.0442 |
| ADt | 199 | (-56, 28, 8) | Inferior Frontal Gyrus, pars triangularis; Frontal Pole; Middle Frontal Gyrus | HTN > HC | 0.0010 ± 0.0001 | 0.0008 ± 0.0001 | 0.0002 (0.0001, 0.0002) | 1.303 | 4.048 | 0.0480 |
| ADt | 242 | (68, -30, 0) | Middle Temporal Gyrus, posterior division; Superior Temporal Gyrus, posterior division; Middle Temporal Gyrus, temporooccipital part | HTN > HC | 0.0009 ± 0.0001 | 0.0008 ± 0.0001 | 0.0001 (0.0000, 0.0001) | 1.044 | 3.134 | 0.0490 |
| ADt | 267 | (-38, 57, 11) | Frontal Pole | HTN > HC | 0.0009 ± 0.0002 | 0.0008 ± 0.0001 | 0.0001 (0.0001, 0.0002) | 1.046 | 3.362 | 0.0486 |
| ADt | 560 | (-21, 4, -14) | Frontal Orbital Cortex; Insular Cortex; Temporal Pole | HTN > HC | 0.0009 ± 0.0001 | 0.0007 ± 0.0001 | 0.0001 (0.0001, 0.0002) | 1.161 | 4.343 | 0.0428 |
| ADt | 878 | (35, -93, 10) | Occipital Pole; Lateral Occipital Cortex, superior division; Lateral Occipital Cortex, inferior division | HTN > HC | 0.0010 ± 0.0001 | 0.0008 ± 0.0001 | 0.0002 (0.0001, 0.0002) | 1.512 | 4.687 | 0.0418 |
| ADt | 3946 | (-18, 59, 28) | Frontal Pole; Middle Frontal Gyrus; Superior Frontal Gyrus | HTN > HC | 0.0010 ± 0.0001 | 0.0008 ± 0.0001 | 0.0001 (0.0001, 0.0002) | 1.662 | 4.599 | 0.0306 |
| ADt | 4262 | (-54, 1, 44) | Insular Cortex; Precentral Gyrus; Central Opercular Cortex | HTN > HC | 0.0010 ± 0.0001 | 0.0009 ± 0.0001 | 0.0001 (0.0001, 0.0002) | 1.533 | 4.360 | 0.0336 |
| ADt | 19712 | (35, 20, -5) | Frontal Pole; Temporal Pole; Precentral Gyrus | HTN > HC | 0.0009 ± 0.0001 | 0.0008 ± 0.0001 | 0.0001 (0.0001, 0.0002) | 1.880 | 4.959 | 0.0146 |
| ADt | 32379 | (-21, -54, -18) | Precuneous Cortex; Cingulate Gyrus, posterior division; Lateral Occipital Cortex, superior division | HTN > HC | 0.0009 ± 0.0001 | 0.0008 ± 0.0001 | 0.0001 (0.0001, 0.0001) | 1.590 | 5.599 | 0.0180 |
| RDt | 75 | (60, -43, -24) | Inferior Temporal Gyrus, temporooccipital part; Inferior Temporal Gyrus, posterior division; Middle Temporal Gyrus, posterior division | HTN > HC | 0.0005 ± 0.0001 | 0.0004 ± 0.0001 | 0.0001 (0.0001, 0.0002) | 1.096 | 3.537 | 0.0486 |
| RDt | 3265 | (35, -93, 10) | Lateral Occipital Cortex, inferior division; Occipital Pole; Lateral Occipital Cortex, superior division | HTN > HC | 0.0007 ± 0.0001 | 0.0006 ± 0.0001 | 0.0001 (0.0001, 0.0001) | 1.517 | 4.500 | 0.0362 |
| RDt | 95379 | (-21, -54, -19) | Frontal Pole; Middle Frontal Gyrus; Precuneous Cortex | HTN > HC | 0.0007 ± 0.0001 | 0.0006 ± 0.0000 | 0.0001 (0.0001, 0.0001) | 1.952 | 5.223 | 0.0034 |

**Table S3. Voxel-wise associations between systolic blood pressure and diffusion metrics in the HTN group**

*All listed associations were negative and survived TFCE FWER correction. Cluster means are HTN-only descriptive values (n=30). HC values, group effect sizes, and group-difference confidence intervals are not applicable to a within-HTN SBP association analysis.*

| Tissue | Metric | Cluster (voxels) | Peak MNI (x, y, z) | Anatomical labels | Direction | HTN mean ± SD | Peak statistic | Min FWER P |
| --- | --- | --- | --- | --- | --- | --- | --- | --- |
| WM | FA | 1 | (-26, -57, 19) | Unclassified | Higher SBP -> lower metric | 0.7121 ± 0.0456 | 3.294 | 0.0500 |
| WM | FA | 6 | (-19, -50, 50) | Unclassified | Higher SBP -> lower metric | 0.5180 ± 0.0645 | 4.257 | 0.0496 |
| WM | FA | 17 | (-26, -70, 13) | Unclassified | Higher SBP -> lower metric | 0.6630 ± 0.0459 | 3.713 | 0.0492 |
| WM | FA | 19 | (17, -59, 48) | Unclassified | Higher SBP -> lower metric | 0.5120 ± 0.0690 | 5.952 | 0.0478 |
| WM | FA | 37 | (-26, -60, 13) | Posterior thalamic radiation (include optic radiation) L | Higher SBP -> lower metric | 0.8166 ± 0.0563 | 3.319 | 0.0486 |
| WM | FA | 58 | (20, 31, 20) | Anterior corona radiata R | Higher SBP -> lower metric | 0.4852 ± 0.0558 | 2.549 | 0.0496 |
| WM | FA | 309 | (18, 5, 37) | Body of corpus callosum; Superior corona radiata R; Genu of corpus callosum | Higher SBP -> lower metric | 0.6647 ± 0.0395 | 3.942 | 0.0470 |
| WM | FA | 325 | (-11, 10, 26) | Body of corpus callosum; Anterior corona radiata L; Genu of corpus callosum | Higher SBP -> lower metric | 0.6150 ± 0.0494 | 3.499 | 0.0458 |
| WM | FA | 344 | (4, 30, 3) | Genu of corpus callosum; Body of corpus callosum | Higher SBP -> lower metric | 0.7690 ± 0.0383 | 4.289 | 0.0456 |
| WM | FA | 1790 | (26, -53, 17) | Body of corpus callosum; Splenium of corpus callosum; Posterior corona radiata R | Higher SBP -> lower metric | 0.7030 ± 0.0331 | 5.223 | 0.0368 |
| GM | FA | 2 | (-23, -49, -11) | Temporal Occipital Fusiform Cortex; Lingual Gyrus; Temporal Fusiform Cortex, posterior division | Higher SBP -> lower metric | 0.1091 ± 0.0341 | 2.294 | 0.0500 |
| GM | FA | 18 | (-18, -49, -11) | Lingual Gyrus; Temporal Occipital Fusiform Cortex; Temporal Fusiform Cortex, posterior division | Higher SBP -> lower metric | 0.1139 ± 0.0306 | 3.187 | 0.0496 |
| GM | FA | 54 | (-30, -83, -20) | Lateral Occipital Cortex, inferior division; Occipital Fusiform Gyrus; Occipital Pole | Higher SBP -> lower metric | 0.0930 ± 0.0303 | 3.758 | 0.0490 |
| GM | FA | 169 | (-29, -55, -7) | Occipital Fusiform Gyrus; Lingual Gyrus; Temporal Occipital Fusiform Cortex | Higher SBP -> lower metric | 0.1049 ± 0.0207 | 5.147 | 0.0432 |
| GM | FA | 2399 | (-40, -79, -35) | Inferior Temporal Gyrus, temporooccipital part; Temporal Occipital Fusiform Cortex; Occipital Fusiform Gyrus | Higher SBP -> lower metric | 0.1483 ± 0.0213 | 4.481 | 0.0236 |
| GM | FAt | 5737 | (-29, -55, -7) | Lingual Gyrus; Occipital Fusiform Gyrus; Temporal Occipital Fusiform Cortex | Higher SBP -> lower metric | 0.2224 ± 0.0368 | 4.786 | 0.0222 |
